# Validation and Factor Analysis of the Obsessive Compulsive Drinking Scale (OCDS) in the Chinese Population

**DOI:** 10.3389/fpsyt.2021.770860

**Published:** 2021-12-02

**Authors:** Hongxuan Wang, Lihuan Lan, Xiaochang Lan, Peiyun Chen, Gaoxin Liu, Xiaoming Rong, Lei Liu, Chuan-Yi Kang, Jianzhong Yang, Yan-Zhong Guan, Xiao-Feng Zhu, Jian Hu, Mei Yang, Dong Zheng, Ying Peng

**Affiliations:** ^1^Department of Neurology, Sun Yat-sen Memorial Hospital, Sun Yat-sen University, Guangzhou, China; ^2^The Affiliated Brain Hospital of Guangzhou Medical University, Guangzhou, China; ^3^Mental Health Centre, First Affiliated Hospital of Harbin Medical University, Harbin, China; ^4^Department of Psychiatry, The Second Affiliated Hospital of Kunming Medical University, Kunming, China; ^5^Department of Physiology and Neurobiology, Mudanjiang Medical University, Mudanjiang, China; ^6^Addiction Medicine Department, Shenzhen Mental Health Center, Shenzhen Kangning Hospital, Shenzhen, China; ^7^Department of Neurology, The Affiliated Brain Hospital of Guangzhou Medical University, Guangzhou, China

**Keywords:** Chinese, Obsessive Compulsive Drinking Scale, OCDS, validation, factor analysis

## Abstract

Obsessive Compulsive Drinking Scale (OCDS) was established and introduced to measure the craving for alcohol and the severity of alcohol dependence. However, the Chinese version of OCDS is still unavailable and has not been validated in the Chinese population. We tended to translate and validate the OCDS in Chinese. We translated original OCDS into Chinese through bi-direction translations and tested the reliability and validity. We found that Chinese OCDS had high internal consistency and good test-retest reliability. The Chinese OCDS also presented good internal structure to reflect the severity of alcohol dependence. The Chinese OCDS could be used in clinical studies and research among the Chinese population.

## Introduction

Excessive alcohol use is one of the most common unhealthy habits worldwide, which is a major risk factor for death and disability. WHO's newest report showed that more than 3 million global deaths are attributed to alcohol use (1). It is also a critical social and health issue in China, since alcohol consumption in China has been increasing for decades (2). It is urgent to control harmful use of alcohol to reduce the national disease burden in China.

Alcohol dependence (AD) is the severer pattern of alcohol use disorders, which is characterized with the strong internal drive to use alcohol without control. Since there were very limited therapies to show sufficient efficacy to prevent the incidents and relapses of alcohol dependence, many clinical scientists are still making tremendous efforts to explore novel effective therapies for alcohol dependence.

Craving for alcohol is the characteristic feature to represent the strong internal drive to alcohol consumption. It is crucial to assess patients' craving for alcohol in the clinical trials to measure and compare the effects of different therapies (3). Several ways have been established to measure the craving for alcohol (4), and the Obsessive Compulsive Drinking Scale (OCDS) was established and introduced to clinical assessment and research by Raymond F. Anton in 1995 (5, 6). It had been validated and widely used in many clinical trials of alcohol use disorders (7–12). Furthermore, OCDS has been translated and validated in different languages (13–15). It showed excellent reliability and validity to measure craving for alcohol.

However, the OCDS is still unavailable in the Chinese population since the Chinese-translated version has not been published and validated yet. In this study, we translated the original OCDS into Chinese through bi-direction translations and performed several tests to analyze its reliability and validity. We found that the Chinese version of OCDS was appropriate to be applied in regular clinical research and it would be effective in self-reported measurements of craving for alcohol in the Chinese population.

## Methods

### Translation

The original OCDS was translated into a Chinese version by forward-backward translations. First, three clinicians translated English OCDS items into Chinese by forward translation independently. Discrepancies were compared and evaluated by a fourth clinician and the most appropriate translated expressions were decided to be used in the forward translation of OCDS. Second, another two investigators who were familiar with Chinese and English cultures and languages translated the Chinese OCDS into English backward. The English expressions in the backward translation were compared to those in the original English version, and the discrepancies of expressions of words or sentences were compared, analyzed, and discussed with consideration of different cultures and concepts. Third, the Chinese expressions in the forward translation were revised into the most appropriate expressions according to comparison. Finally, the Chinese OCDS was tested in a preliminary sample of patients and healthy volunteers, and some expressions of the items in the scale were further revised until all expressions in the translated scale were reported to be clear enough for reading and understanding by Chinese-speaking participants. Then, the final version of Chinese OCDS was established and ready for further assessments of reliability and validity.

### Participants

Patients were enrolled from three different clinic centers: Sun Yat-sen Memorial Hospital of Sun Yat-sen University, the Affiliated Brain Hospital of Guangzhou Medical University, and Shenzhen Kangning Hospital. The study protocol was approved by the ethic committee of Sun Yat-sen Memorial Hospital, Sun Yat-sen University. Patients who met the DSM-4 diagnostic criteria for alcohol dependence and aged from 18 to 65 years old were included. Patients with the following conditions were excluded in our study: patients who had acute withdrawal symptoms; patients who were addicted to other substances; patients who had severe mental illness such as major depression, schizophrenia, schizophreniform psychosis; patients who had acute brain injuries, stroke, encephalitis, or other acute central nervous system diseases; patients who had functional disabilities which impeded them from communicating well with others by speech or words; patients who had moderate-to-severe cognitive impairment or dementia; and patients who were reluctant to complete the examinations. All the patients were requested to participate in the assessment of the Chinese version of OCDS and other scales, and the patients repeated a second assessment with the Chinese version of OCDS again later. The test-retest procedures were all self-reported assessments with minimal help of the researchers and were performed with the interval of 24 hours.

### Measurements

#### OCDS

The OCDS is composed of 14 items, and each item has a 5-point response (from 0 to 4 by self-report) (5). The total score of 14 items ranges from 0 to 56. In Anton's designed setting (5), four pairs of original items were calculated into four adjusted scores: the higher score between items 1 and 2, the higher score between items 7 and 8, the higher score between items 9 and 10, and the higher score between items 13 and 14 were identified as representing scores of the item pairs, respectively. Therefore, the adjusted total score was calculated by adding up 10 of all 14 items together, including 6 original item scores (item 3 to 6, item 11, item 12) and four adjusted item scores from the four pairs of original items (item pair 1 and 2, item pair 7 and 8, item pair 9 and 10, and item pair 13 and 14). The adjusted total score ranges from 0 to 40, and the higher total score indicates more obsessive thoughts or compulsive behaviors regarding alcohol use.

Moreover, the adjusted total OCDS score can be divided into two subscales in Anton's setting (5): the obsessive thoughts subscale (OB, the sum of adjusted item pair 1/2 and items 3 to 6, which is composed of five 5-point scores together and ranges from 0 to 20) and the compulsive drinking subscale (CP, the sum of adjusted item pair 7/8, 9/10, 13/14, and items 11 to 12, which is also composed of five 5-point scores together and ranges from 0 to 20).

#### Other Measurements for Alcohol Dependence or Alcohol Use Disorders

The Alcohol Dependence Scale (ADS) was originally designed to assess the degree of severity of the alcohol dependence syndrome in 1984 (16, 17). It has been tested to have very good internal consistency and it has been validated in many previous studies (18–20). The total raw score ranges from 0 to 47, where 0 suggests no evidence of alcohol dependence. Total score of 1 to 13, 14 to 21, 22 to 30, or more than 30 suggests a low, intermediate, substantial, or severe level of alcohol dependence, respectively.

The Alcohol Use Disorders Identification Test (AUDIT) was developed specifically to identify alcohol use disorders by World Health Organization (WHO) in the 1980s (21, 22). It has been widely used and translated into different languages, including Chinese. The Chinese versions of AUDIT have been validated in many studies (23). We used AUDIT as a referential scale to identify alcohol dependence and measure the relation between OCDS and AUDIT. The total score of AUDIT ranges from 0 to 40, where 0 suggests no evidence of an alcohol drinking problem. Total score of 1–7 suggests low-risk alcohol consumption, while 8–14 or more than 15 suggests hazardous/harmful alcohol consumption or alcohol dependence (moderate-severe alcohol use disorder), respectively.

Visual analog scale (VAS) was also used to measure the participants' craving for alcohol. VAS ranges from 0 to 10, where 0 means no craving and 10 means most craving or strongest drive to consume alcohol.

### Statistics

Internal consistency of the scale was assessed by Cronbach α. The test-retest reliability was measured by Pearson correlation. Correlations among items, correlations between each item/dimension and the total/subtotal scale, and correlations between different scales were analyzed by Pearson correlation analysis.

Content validity was measured by construct validity analysis and concurrent validity analysis. Construct validity of the translated OCDS was tested by exploratory factor analysis and confirmatory factor analysis. Concurrent validity was conducted through multiple correlation between different items or factors with other scales.

The analysis was conducted by the R version 3.6.0. In the factor analysis, package Psych for R was used to perform the exploratory factor analysis (EFA) and package Lavaan for R was used for the confirmatory factor analysis (CFA). Briefly, in EFA, correlation matrix of the OCDS data was used to identified optimal numbers of factors by Horn's parallel analysis in the package Psych for R (https://cran.r-project.org/web/packages/psych/index.html). Then factor analysis was performed based on the optimal number of factors and different methods of factor rotation including none rotation, varimax rotation, and promax rotation were compared and identified. The model fitness was measured by factor loadings from the pattern matrix, proportion variance of each factor, and cumulative variance. In the CFA package Lavaan for R was used to calculate the fitness statistics in the test model of factor analysis (https://cran.r-project.org/web/packages/lavaan/index.html) (24), and goodness-of-fit index (GFI), non-normed fit index (NNFI) and comparative fit index (CFI) was used for assessing the fitness of the model.

## Results

A total of 87 patients with alcohol dependence was recruited, and 83 patients of them were male (95.4%). The patients aged from 22 to 65 years old, and the mean age of the patients was 42.12 ± 11.33 years old. In this group of participants, their mean ADS was 12.29 ± 8.47, and mean AUDIT was 21.07 ± 7.88 (Table 1).

**Table 1:** Characteristics of the participants and internal consistency of measurements.

**Characteristic**	**Total subjects**	
N	87	
Male:female	83:4	
Age (years)	42.12 ± 11.33	
		Internal consistency (α)
ADS	12.29 ± 8.47	0.86
AUDIT	21.07 ± 7.88	0.75
AUDIT-C	8.11 ± 3.21	0.76
VAS (range, quartile)	0–10 (0, 3)	/
Original total OCDS	23.69 ± 12.36	0.90
Adjusted total OCDS	17.39 ± 9.19	0.87
Original total re-test OCDS	20.61 ± 12.15	0.91
Adjusted total re-test OCDS	15.56 ± 9.03	0.88

*ADS, Alcohol Dependence Scale; AUDIT, Alcohol Use Disorders Identification Test; AUDIT-C, Alcohol Use Disorders Identification Test Consumption; VAS, Visual Analog Scale; OCDS, Obsessive Compulsive Drinking Scale*.

The total score of the original OCDS ranged from 0 to 55, and the median score was 22.5, and the mean score was 23.69 ± 12.36. The adjusted total OCDS ranged from 0 to 40, and the median score was 17, and the mean score was 17.39 ± 9.19 (Table 1).

For all 14 items in OCDS, the internal consistency of the Chinese version of OCDS was excellent with the Chronbach's α showing 0.90 for the first tests and 0.91 for the retests. For the adjusted OCDS including 10 scoring items, the internal consistency of the OCDS was 0.87 for the first tests and 0.88 for the retests. The internal consistency of OCDS was as excellent as other scales (Table 1). The test-retest reliability was good for both original and adjusted OCDS (*r* = 0.72, *p* < 0.001).

Then, we tested the structure of the OCDS and measured the construct validity by factor analysis. First, we used exploratory factor analysis (EFA) to find that there were 3 factors which would well-explain the internal structure of the Chinese OCDS with all original items, while there were two factors which well-fit the 10-item adjusted OCDS. In order to compare with previous studies, data from 14 original items were used in further factor analysis and comparison, because previous studies only included original items into factor analysis. For original items of OCDS, we subjected the factors by varimax rotation in the EFA and found out the three factors accounting for 68% of the variation in our Chinese OCDS item scores. Factor 1 indicated the resistance to the alcohol consumption, with original items 5, 6, 11, 12, 13, and 14 loading on it. Factor 2 measured the interference from the alcohol drinking problems, and it included original items 3, 4, 9, and 10. Factor 3 reflected obsessions and compulsions for alcohol, and it consisted of original items 1, 2, 7, and 8 (Table 2).

**Table 2:** Factor loadings from the pattern matrix and percent variance explained by each factor.

**Factor**	**Item**	**Factor pattern**			**Comm**
		**F1**	**F2**	**F3**	
Factor 1	Resistance to the alcohol consumption (Explained 39%)				
	5	**0.81**	0.03	0.28	1.2
	6	**0.73**	0.13	0.27	1.3
	11	**0.60**	0.35	0.03	1.6
	12	**0.77**	0.06	0.13	1.1
	13	**0.83**	0.23	0.02	1.2
	14	**0.70**	0.22	0.19	1.4
Factor 2	Interference from the alcohol drinking problems (Explained 38%)				
	3	0.19	**0.93**	0.09	1.1
	4	0.22	**0.85**	0.09	1.2
	9	0.11	**0.90**	0.19	1.1
	10	0.20	**0.91**	0.11	1.1
Factor 3	Obsessions and compulsions for alcohol (Explained 23%)				
	1	0.32	0.32	**0.60**	2.1
	2	0.27	0.30	**0.65**	1.8
	7	0.00	0.02	**0.67**	1.0
	8	0.23	0.00	**0.83**	1.2

*Comm, complexity*.

Second, we used confirmatory factor analysis (CFA) to verify the internal structures of the Chinese OCDS and to compare our EFA model with the other previous reported models. The results of different CFA models are shown in Table 3. Comparing to other models in previous studies, our new 3-factor model from EFA showed good fitness to the Chinese OCDS (CFI was 0.87, which nearly achieved optimal fitness as 0.90), and the identified factors were consistent with some models from previous studies, such as Connor's 4-factor model (12) and Bohn's 4-factor model (7). Original items 3, 4, 9, and 10 were fit for one of the four factors identified by Connor et al., while original items 5, 6, 11, 12, 13, and 14 were fit for another one of the four factors identified by Bohn et al.

**Table 3:** Measures of fit for different models of internal structure of OCDS.

**Test models**	**Absolute**			**Relative**		
	**χ^2^**	**df**	***P***	**GFI**	**NNFI**	**CFI**
new EFA 3-factor model	195.62	74	<0.001	0.75	0.85	0.87
Anton's 2-factor model (5, 6)	292.11	34	<0.001	0.60	0.37	0.52
Kranzler's 3-factor model (9)	493.77	74	<0.001	0.50	0.47	0.57
Robert's 3-factor model (10)	331.68	74	<0.001	0.66	0.67	0.73
Bohn's 4-factor model (7)	265.68	71	<0.001	0.72	0.74	0.80
Connor's 4-factor model (11)	316.48	71	<0.001	0.69	0.67	0.75
Connor's 4-factor model 2 (12)	190.95	71	<0.001	0.80	0.84	0.88

*GFI, goodness-of-fit index; NNFI, non-normed fit index; CFI, comparative fit index*.

Finally, we tested concurrent validity by correlations between OCDS and other scales measuring alcohol dependence. For the Chinese OCDS, the correlation of the original total OCDS with the Chinese version of ADS was moderate to high (*r* = 0.47, *p* < 0.001), while the adjusted total OCDS was also correlated tightly with ADS (*r* = 0.47, *p* < 0.001). The correlation of the original total OCDS with the Chinese version of AUDIT was good (*r* = 0.71, *p* < 0.001), and similar correlation was also found between adjusted total OCDS and AUDIT (*r* = 0.68, *p* < 0.001). The correlation of total OCDS with the Chinese version of AUDIT-C was mild but significant (*r* = 0.31, *p* = 0.005), and so as the correlation of the adjusted total OCDS with AUDIT-C (*r* = 0.27, *p* = 0.011). However, both total and adjusted Chinese-translated OCDS were not correlated with VAS (*r* = 0.14 and *r* = 0.18, respectively, *p* > 0.05). In our model of factor analysis, all the three factors of the Chinese-translated OCDS correlated with ADS and AUDIT well (all *p* < 0.005). The correlations between each factor and different scales were shown in Table 4. Although the total OCDS was not correlated with VAS, factor 3 of the OCDS correlated with AUDIT-C and VAS (*p* < 0.001 and *p* < 0.005, respectively).

**Table 4:** Concurrent validities of OCDS and its factors with other scales.

**OCDS scores**	**OCDS**	**ADS**	**AUDIT**	**AUDIT-C**	**VAS**
Original total score	1.00*	0.47*	0.71*	0.31	0.14
Adjusted total score	0.99*	0.47*	0.68*	0.27^†^	0.18
Anton's 2-factor model					
Obsessive subscale	0.96*	0.47*	0.62*	0.24^†^	0.14
Compulsive subscale	0.94*	0.44*	0.67*	0.30^#^	0.15
Connor's 4-factor model 2					
F1	0.83*	0.45*	0.58*	0.09	−0.05
F2	0.73*	0.47*	0.68*	0.33^‡^	0.36*
F3	0.50*	0.09	0.56*	0.79*	0.14
F4	0.82*	0.31^‡^	0.44*	0.13	0.15
The new 3-factor model					
F1	0.84*	0.33^‡^	0.48*	0.13	0.18
F2	0.75*	0.43*	0.53*	0.08	−0.12
F3	0.72*	0.34^‡^	0.72*	0.62*	0.30^‡^

* *p < 0.001*;

‡ *p < 0.005*;

# *p < 0.01*;

† *p < 0.05*.

*ADS, Alcohol Dependence Scale; AUDIT, Alcohol Use Disorders Identification Test; AUDIT-C, Alcohol Use Disorders Identification Test Consumption; VAS, Visual Analog Scale; OCDS, Obsessive Compulsive Drinking Scale*.

## Discussion

Obsessive Compulsive Drinking Scale (OCDS) was a self-report scale to measure craving of the patients with alcohol dependence. It has been used in many studies and have been validated in several languages. However, the Chinese version and its validity has not been published yet. In this study, we translated the original OCDS into Chinese through the bi-direction translation method. After we recruited patients to test the reliability and validity, we found that the Chinese version of OCDS had high internal consistency and good test-retest reliability for measuring the severity of alcohol dependence. Our results showed that the Chinese version of OCDS could measure similar conditions stably for individual patients within a short period, and the results also showed a well-co-related structure among different items. Therefore, it acted as a good tool for screening patients with alcohol dependence in clinical daily use and trials.

For the internal structure of Chinese OCDS, several strategies were compared to identify the factors among all items of the whole scale. We found that the three-factor model could well-explain the structure of the scale (three factors accounting for 68% of the variation). Both our new model and Connor's model (12) indicated that original items 3, 4, 9, and 10 in OCDS reflected the structure measuring interference from alcohol, while both our new model and Bohn's model (7) suggested original items 5, 6, 11, 12, 13, and 14 in OCDS reflected the structure measuring resistance to alcohol consumption. In our model, items 1, 2, 7, and 8 were grouped into the same factor, which reflected obsessions and compulsions for alcohol. This was due to the tight correlation among these four items, which represented the craving for alcohol, the intention in the patients' minds and efforts to consume alcohol. These four items could be further grouped into two separate factors reflecting obsession and compulsion, respectively (1 and 2 for obsessions; 7 and 8 for compulsion) in Connor's study (12).

In the CFA, although the goodness-of-fit index (GFI) of the 3-factor model in the Chinese OCDS was 0.75 and did not achieve optimal fit (GFI > 0.90), the GFI was similar to the GFIs calculated in the previous studies with Connor's 4-factor model and Bohn's 4-factor model. The GFI of the Chinese OCDS calculated through Connor's 4-factor model and Bohn's 4-factor were 0.80 and 0.72, respectively, which were comparable to the previously reported GFIs of the validated OCDS in both Connor's and Bohn's works (0.79 and 0.86, respectively). Therefore, OCDS could reflect different aspects of the craving for alcohol or the severity of alcohol dependence. The Chinese OCDS has similar internal structures as previous reports from other countries or population in several languages (7, 9–12).

Our preliminary data also suggested that the total score of OCDS and its internal structure would reflect clinical severity of alcohol dependence measured by ADS or clinical diagnostic features of alcohol dependence measured by AUDIT, and the factor 3 or its composed items would also reflect the severity of alcohol dependence. Our results revealed that the Chinese version of OCDS had enough reliability and proved validity in clinical and research use for Chinese populations.

However, our study also had some limitations. First, small sample size was a limitation for our study. Our sample size was only slightly more than the earliest study by Anton (5). However, our patients were enrolled from different hospitals or centers of different regions in China, which prevented our data from selected bias to some extent. In addition, the analysis and the results with the data supported the reliability and validity of the Chinese version of OCDS. Second, the Alcohol Dependence Scale (ADS) was designed to measure the severity of alcohol drinking problems. Although the Chinese version of ADS can be found in many Chinese publications and the translation was uniform, there is no published validation study of ADS in the literature yet. No other validated Chinese scale measuring the severity of alcohol dependence is available, we still used ADS as one of the reference scales in the correlation analysis with OCDS. Third, we had not tested the reliability and validity in different subgroups of patients with alcohol dependence stratified by age, different ethnics of Chinese, different stages of diseases or comorbidities, and so on. In order to test the reliability and validity among different characterized subgroups, more patients should be included in further study. Moreover, we have not yet known whether there is any confounding factor affecting the reliability and validity, further study is needed for the complex intention. Finally, we had not tested whether baseline OCDS might predict future prognosis for the patients and whether it would have stable and predictable value in the perspective cohort studies. We tend to conduct further studies about the Chinese OCDS in later clinical studies to explore and verify its clinical and research significance for perspective trials.

In conclusion, our results suggested that the Chinese version of OCDS might be important and useful for the trials or research holding in the Chinese population and society. We can use OCDS to measure the severity of alcohol dependence of the Chinese participants in future studies.

## Data Availability Statement

The raw data supporting the conclusions of this article will be made available by the authors, without undue reservation.

## Ethics Statement

The studies involving human participants were reviewed and approved by Sun Yat-sen Memorial Hospital, Sun Yat-sen University. The patients/participants provided their written informed consent to participate in this study.

## Author Contributions

HW and LLa analyzed the data and wrote the manuscript. LLa, XL, PC, GL, XR, LLi, C-YK, Y-ZG, MY, and DZ collected the data. HW, X-FZ, JY, JH, and YP revised the manuscript. HW, JY, JH, MY, and YP designed the study. All authors contributed to the article and approved the submitted version.

## Funding

This work was supported by the funding of National Key R&D Program of China (2018YFC1314400 and 2018YFC1314401 to YP).

## Conflict of Interest

The authors declare that the research was conducted in the absence of any commercial or financial relationships that could be construed as a potential conflict of interest.

## Publisher's Note

All claims expressed in this article are solely those of the authors and do not necessarily represent those of their affiliated organizations, or those of the publisher, the editors and the reviewers. Any product that may be evaluated in this article, or claim that may be made by its manufacturer, is not guaranteed or endorsed by the publisher.
